# Effects of electronic cigarette liquid on monolayer and 3D tissue-engineered models of human gingival mucosa

**DOI:** 10.15171/japid.2019.010

**Published:** 2019-12-18

**Authors:** Zahab N Shaikh, Abdullah Alqahtani, Thafar Almela, Kirsty Franklin, Lobat Tayebi, Keyvan Moharamzadeh

**Affiliations:** ^1^School of Clinical Dentistry, University of Sheffield, Claremont Crescent, Sheffield, S10 2TA, UK; ^2^Marquette University School of Dentistry, Milwaukee, WI 53233, USA

**Keywords:** Electronic cigarette, cytotoxicity, keratinocyte, fibroblast, tissue engineering, wound healing

## Abstract

**Background:**

There is limited data available on potential biological effects of E-cigarettes on human oral tissues. The aim of this study was to evaluate the effects of E-cigarette liquid on the proliferation of normal and cancerous monolayer and 3D models of human oral mucosa and oral wound healing after short-term and medium-term exposure.

**Methods:**

Normal human oral fibroblasts (NOF), immortalized OKF6-TERET-2 human oral keratinocytes, and cancerous TR146 keratinocyte monolayer cultures and 3D tissue engineered oral mucosal models were exposed to different concentrations (0.1%, 1%, 5% and 10%) of E-cigarette liquid (12 mg/ml nicotine) for 1 hour daily for three days and for 7 days. Tissue viability was monitored using the PrestoBlue assay. Wounds were also produced in the middle surface of the monolayer systems vertically using a disposable cell scraper. The alterations in the cell morphology and wound healing were visualized using light microscopy and histological examination.

**Results:**

Statistical analysis showed medium-term exposure of TR146 keratinocytes to 5% and 10% E-liquid concentrations significantly increased the viability of the cancer cells compared to the negative control. Short-term exposure of NOFs to 10% E-liquid significantly reduced the cell viability, whereas medium-term exposure to all E-liquid concentrations significantly reduced the NOF cells’ viability. OKF6 cells exhibited significantly lower viability following short-term and mediumterm exposure to all E-cigarette concentrations compared to the negative control. 3D oral mucosal model containing normal oral fibroblasts and keratinocytes showed significant reduction in tissue viability after exposure to 10% E-liquid, whereas medium-term exposure resulted in significantly lower viability in 5% and 10% concentration groups compared to the negative control. There was a statistically significant difference in wound healing times of both NOF and OKF6 cells after exposure to 1%, 5% and 10% E-cigarette liquid.

**Conclusion:**

Medium-term exposure to high concentrations of the E-cigarette liquid had cytotoxic effects on normal human oral fibroblasts and OKF6 keratinocytes, but a stimulatory cumulative effect on the growth of cancerous TR146 keratinocyte cells as assessed by the PrestoBlue assay and histological evaluation of 3D oral mucosal models. In addition, E-liquid exposure prolonged the wound healing of NOF and OKF6 oral mucosa cells.

## Introduction

Electronic cigarette (EC) use has rapidly increased since they were introduced. There are significant debates in healthcare and regulatory bodies on this subject, and conflicting advice has been given regarding the appropriate use of ECs.

Data from an international survey showed 15% use in the US, 10% use in the UK, 4% use in Canada, and 2% use in Australia and the number of young people trying the ECs is significantly increasing.^1^ The rate of EC use by middle and high school students in the US has tripled from 2011 to 2013. A three-fold increase in EC use was reported from 2010 to 2012 in the Great Britain.^2^

There is a growing body of evidence that demonstrates ECs are an effective aid to smoking cessation, and they have significant potential to reduce tobacco smoke-related oral diseases.^3-5^ However, it is important to balance the potential adverse effects of ECs aerosol with the benefits of reducing tobacco smoking.

Several chemical studies have analyzed the composition of liquids and aerosols from ECs.^6-12^ Several potentially harmful materials have been identified in ECs, including diethylene glycol, propylene glycol, nitrosamines, toluene, acrolein, acetaldehyde, formaldehyde, nickel, lead, aluminum, chromium, cadmium, and silicon. Some ingredients, such as Diacetyl, used in flavored e-liquids are safe to consume orally, though perhaps not to inhale, as it has been associated with bronchiolitis obliterans.

Although a few animal studies have failed to show any obvious adverse systemic toxicity associated with inhaled glycerol or propylene glycol,^13-14^ concerns have been raised for humans in terms of respiratory tract irritation,^15^ and evidence for potential localized adverse effects of inhaled glycerin is sparse. An animal study has shown evidence of mild epithelial squamous metaplasia following long-term inhalation of aerosolized glycerol in rats.^16^

It has been demonstrated that exposure to ECs impairs pulmonary antibacterial and antiviral defenses in a mouse model.^17^ In vitro toxicity profiles of electronic and tobacco cigarettes, smokeless tobacco, and nicotine replacement therapy products have been compared in a study using Ames *Salmonella* mutagenicity testing.^18^

The nicotine in the ECs is mainly absorbed through the buccal mucosa and pharyngeal mucosa. Although nicotine has been shown to have wound healing and angiogenic properties,^19^ which can make it a potential therapeutic agent, there are some concerns regarding its ability to promote lung tumor growth through different possible mechanisms, including angiogenesis, cell proliferation, and cell migration.^20-21^

There are few representative studies that illustrate the negative effects of ECs on the human oral mucosa. Evidence supports that conventional smoking has a negative influence on a patient’s response, as well as their treatment outcome, following non-surgical, surgical, and regenerative periodontal treatment. Smoking also impairs all wound healing phases (particularly the inflammatory and proliferation phases, which lead to delays in wound healing).^22-26^

Clinical studies of ECs have reported mild harmful effects of vaping on selected cardiovascular^27-28^ and respiratory functional outcomes^29-31^ to a considerably less extent when compared with conventional cigarette smoking^32^. However, it is difficult to assess the prognostic implications of these studies, and there is a need for further research in this area.

Clinical research and survey-type studies to date have shown that the most common patient-reported side effects were symptoms such as xerostomia, throat irritation, and cough.^33-36^

A five-year multicenter cohort study was conducted to assess the long-term efficacy and safety of ECs,^37^ and a pilot study investigated the oral mucosa perfusion of intraoral free flaps in EC users and smokers.^38^

Several toxicological studies have been conducted on ECs, using monolayer cell culture systems.^39-43^ However, there is no study published in the literature comparing the effects of E-cigarette liquid on different normal and cancerous oral mucosa cells, including 3D tissue-engineered models of the human oral mucosa. Therefore, this study aimed to evaluate the biological effects of E-cigarette liquid on both full-thickness 3D oral mucosa models and monolayer cell culture systems utilizing normal oral fibroblasts, OKF6-TERET2 oral keratinocytes, and cancerous TR146 keratinocytes, following short-term (three days) and medium-term (seven days) exposure to flavorless electronic cigarette liquid. Additionally, this study aimed to investigate the influence of E-cigarette liquid on oral mucosa wound healing, using normal oral fibroblasts and OKF6 oral keratinocyte cell cultures.

## Methods

### Cell Source and Biological Systems

Normal human oral fibroblast cells were obtained from the stocks stored in liquid nitrogen in the laboratories of the School of Clinical Dentistry at the University of Sheffield. These cells had been previously obtained from healthy patients undergoing oral surgery at Charles Clifford Dental Hospital with their written informed consent under appropriate ethical approval from the UK National Research Ethics Services Committee.

The immortalized OKF6/TERT-2 human oral keratinocyte cell line was kindly provided by Brigham and Women's Hospital, Harvard Institute of Medicine, USA.

The cancerous human TR146 cell line was derived from a neck lymph node metastasis originating from a carcinoma of the oral buccal mucosa. These cells were kindly provided by Cancer Research UK.

Five different biological systems were tested in this study, including:

- Monolayer cultures of normal oral fibroblasts (NOF)
- Monolayer cultures of the immortalized oral keratinocyte cell line (OKF6/TERT-2)
- Monolayer cultures of cancerous TR146 keratinocytes
- Co-cultures of NOF and OKF6/TERT-2 cells
- 3D tissue-engineered oral mucosa models using TR146 keratinocytes and NOFs

### Cell Culture

Oral fibroblasts and keratinocytes were cultured in high-glucose Dulbecco’s modified Eagle’s medium (DMEM) (Sigma, UK), supplemented with 2% L-glutamine (Sigma, UK), 100 IU:100 mg ml-1 Penicillin/Streptomycin (Sigma, UK), and 10% fetal calf serum (FCS) (Biowest Ltd., UK). Six-well plates were utilized for the monolayer cell cultures, inoculating 10,000 cells per well for NOFs, OKF6/TERT2 cells, and TR146 keratinocytes. Additionally, normal oral fibroblasts and keratinocytes were cultured together using six-well plates.

The cultures were maintained in incubators at 5% CO_2_ at 37°C. The cells were cultured until 80‒100% confluency was achieved.

### Tissue-engineered 3D Oral Mucosa Models

Full-thickness 3D tissue-engineered oral mucosa models were manufactured by air/liquid interface culture of TR146 keratinocytes seeded onto fibroblast-populated collagen gels. A solution of 10 × DMEM, 8.5% (v/v) FBS, 2 mM L-glutamine, reconstitution buffer (22 mg mL^−1^ sodium bicarbonate and 20 mM 4-(2-hydroxyethyl)-1-piperazineethanesulfonic acid), and 5 mg mL-^1^ rat tail type I collagen (R & D system, UK) was prepared and neutralized by 1-M sodium hydroxide to pH=7.4 in an ice-cold environment by keeping everything on ice. Normal oral fibroblasts were added to the solution at a concentration of 500,000 cells/model, and 1 mL of the resultant cell-containing collagen mixture was transferred to cell culture transwell inserts (0.4 µm pore size, Millipore), incubated at 37°C for 2 hours until solidified, and then completely submerged in complete DMEM for 3 days. Subsequently, 1×10^6^ keratinocytes were seeded onto each model and kept in submerged culture for three days, after which the oral mucosal models were raised to air/liquid interface and cultured for a further 7 days.

### Exposure Protocol

Neutral E-Cigarette Liquid (Vype-UK) with no added flavors, containing medium nicotine level of 12 mg/mL, was used in this experiment. Four different concentrations (0.1%, 1%, 5%, and 10%) of E-cigarette liquid were prepared by diluting the E-liquid with DMEM.

The monolayer cell culture systems and the 3D oral mucosa model’s epithelial surfaces were exposed to four different concentrations of E-cigarette liquid (0.1%, 1%, 5%, and 10%), to the negative control (DMEM) and the positive control (70% ethanol). The groups consisted of six samples each (n=6).

All the samples were exposed to their respective reagents for one hour daily for three continuous days and incubated at 5% CO_2_ and 37ºC. Following the testing for the short-term exposure, the samples were washed twice with phosphate-buffered saline (PBS) (Sigma, UK), and the medium-term exposure continued for four more days by exposing the samples to the reagents for one hour daily, totaling seven days.

### Cell Viability Assay

Following the short-term and medium-term exposures, tissue viability test was carried out using the PrestoBlue assay. The PrestoBlue reagent (Biosource, Camarillo, CA) was added to the samples at a ratio of 9:1 (volume of cells and culture medium: volume of PrestoBlue reagent). The plates were then incubated for 60 minutes at 37°C and 5% CO_2_. Following incubation, triplicate 200-mL samples were placed into the wells of a 96-well plate, and the fluorescence intensity of each well was measured at an excitation wavelength of 530 nm and an emission wavelength of 590 nm using a fluorescent plate reader (Infinite 200 PRO TECAN, Switzerland).

### Histological and Morphological Assessment

The oral mucosa model samples were first fixed in 10% formalin solution for 24 hours; the samples were then mounted in Optimal Cutting Temperature (OCT) embedding compound, followed by freezing at -20 to -80°C. Afterward, the samples were sectioned at a thickness of 10‒30 μm using a cryostat machine. The sections were then mounted on the histological slides. This was followed by drying the slides for around 30 minutes at room temperature. The slides were then stored in the freezer at -80°C until the processing for hematoxylin and eosin (H&E) staining.

The slides were examined under a light microscope by more than one histopathology expert to assess the changes in the connective tissue and the epithelial layers of the model following the exposure to test agents. Assessment criteria included the continuity and thickness of the epithelium, cell morphology, presence or absence of pyknotic nuclei, presence of a distinct interface between the epithelium and the connective tissue layer.

### Wound Healing Assay

Monolayer cultures of normal oral fibroblasts and OKF6/TERT-2 keratinocytes were developed in 6-well tissue culture plates and divided into negative control (DMEM), positive control (70% ethanol), and test groups with various E-Cigarette concentrations (0.1%, 1%, 5%, and 10%) (n=6). The wounds were produced vertically in the middle of the surface of the monolayer systems using a disposable cell scraper. The test groups were exposed to the culture media containing E-cigarette liquid immediately before and continued daily after creating a wound, and then the wounds were monitored until complete healing had occurred. Microscopic images were obtained pre- and post-wound creation daily to assess the healing time in all the groups.

### Statistical Analysis

SPSS 20 was used for statistical analysis. The normality of the data was analyzed using the normality test (Shapiro-Wilk test). Means of the samples were compared with ANOVA, followed by multiple comparisons using post hoc Tukey tests to determine the differences between the different groups. The level of significance for all the statistical tests was set at a=0.05.

## Results

**Figure 1:**
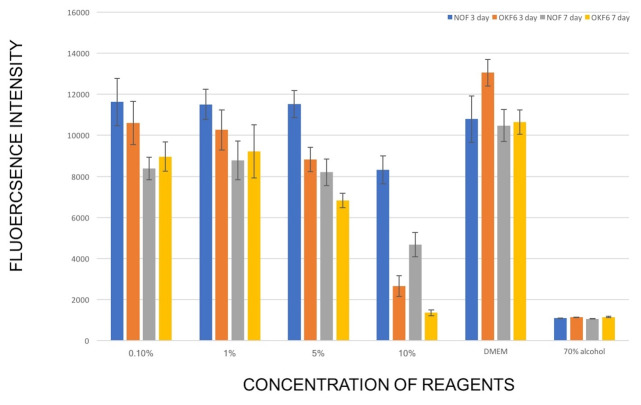
Tissue viability of normal oral fibroblasts and OKF6 keratinocyte monolayer cell cultures exposed to different concentrations of E-liquid as assessed by the PrestoBlue assay.

The results of the PrestoBlue assay for normal oral fibroblasts and OKF6/TERT-2 keratinocyte monolayer cell cultures exposed to different concentrations of the E-Liquid for three days and seven days are shown in Figure 1. Short-term exposure to 10% E-liquid solution caused a statistically significant reduction in the viability of NOFs (P<0.0001) compared to the negative control group. Following medium-term exposure, all the E-liquid concentration groups exhibited significantly lower viability compared to the negative control group.

OKF6/TERT-2 keratinocyte monolayers showed significantly lower viability after short-term exposure to all the E-liquid concentrations, whereas medium-term exposure resulted in significantly lower viability in all the groups except for 1% concentration group compared to the negative control group.

**Figure 2:**
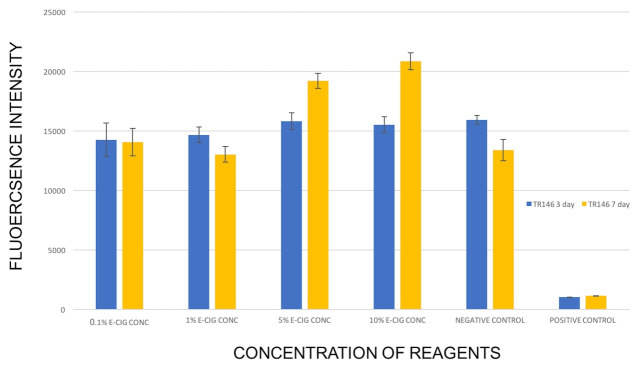
Tissue viability of TR146 keratinocyte monolayer cell cultures exposed to different concentrations of E-liquid as assessed by the PrestoBlue assay.

The TR146 keratinocyte monolayers did not exhibit any statistically significant difference in the viability between different E-liquid concentration groups compared to the negative control group after short-term exposure. However, medium-term exposure to 5% and 10% E-liquid solution caused a statistically significant increase in the viability of TR146 keratinocytes compared to the negative control group (Figure 2).

**Figure 3:**
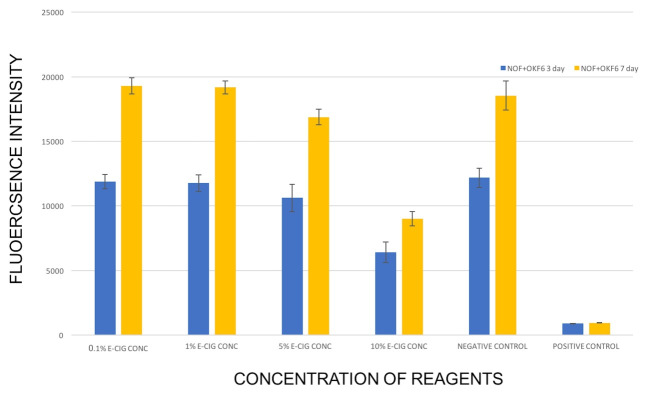
Tissue viability of normal oral fibroblast and OKF6 keratinocyte 3D co-culture systems exposed to different concentrations of E-liquid as assessed by the PrestoBlue assay.

**Figure 4:**
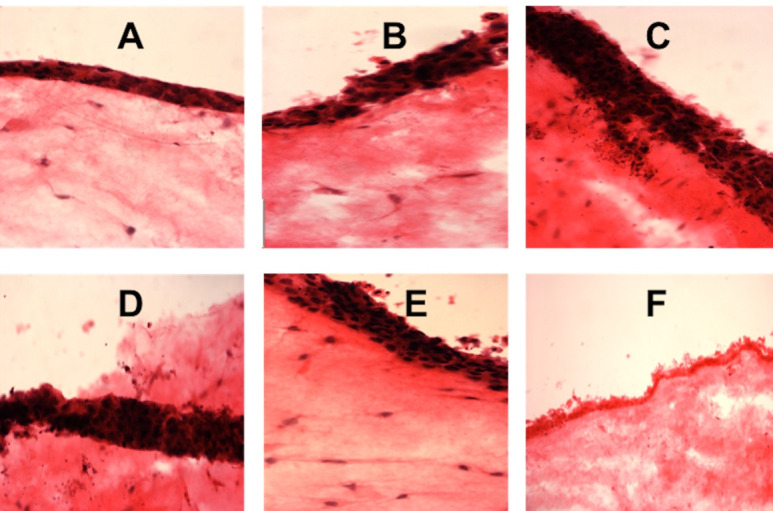
Histological sections of 3D tissue-engineered oral mucosa models after short-term exposure to (A) 0.1% E-liquid; (B) 1% E-liquid; (C) 5% E-liquid; (D) 10% E-liquid; (E) negative control; (F) positive control (H & E staining, original magnification ×10).

**Figure 5:**
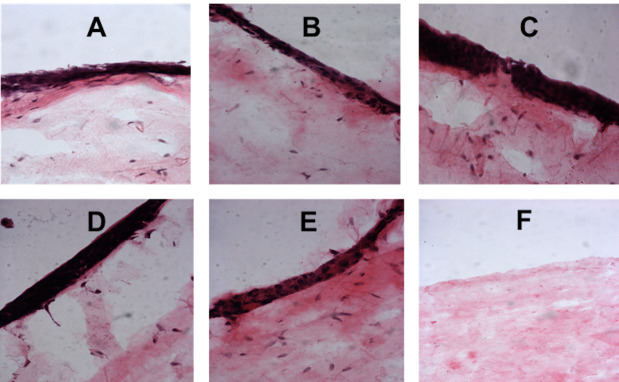
Histological sections of 3D tissue-engineered oral mucosa models after medium-term exposure to (A) 0.1% E-liquid; (B) 1% E-liquid; (C) 5% E-liquid; (D) 10% E-liquid; (E) negative control; (F) positive control (H & E Staining, original magnification ×10).

Normal oral fibroblast and OKF6/TERT-2 keratinocyte co-culture system showed a significant reduction in cell viability in 10% E-liquid concentration group following short-term exposure. In contrast, medium-term exposure resulted in significantly lower viability in 5% and 10% concentration groups (P<0.0001) compared to the negative control group (Figure 3). Histologically, the 3D oral mucosal models utilizing the NOFs and TR146 keratinocytes showed an increase in the thickness of the cancerous epithelial layer in high E-liquid concentration groups compared to the negative control after short-term and medium-term exposure (Figures 4 and 5).

**Figure 6:**
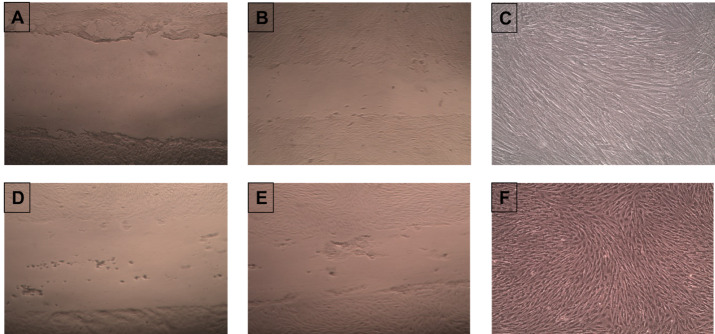
Microscopic views of the wound healing assay showing (A) the center of the wound on day 1 of fibroblast culture; (B) central wound on day 3 of fibroblast culture; (C) completely healed fibroblast cultures; (D) the center of the wound on day 1 of keratinocyte culture; (E) central wound on day 3 of keratinocyte culture; and (F) completely healed keratinocyte cultures.

Figure 6 demonstrates microscopic views of the wound healing at different stages for normal oral fibroblasts and OKF6/TERT-2 keratinocytes. Table 1 presents the mean values and standard deviations of the total time of wound healing for the control and test groups. There was a statistically significant difference in the wound healing time of both NOF and OKF6/TERT-2 monolayer systems exposed to 1%, 5%, and 10% E-liquid solutions compared to those of the negative control group (P<0.05).

**Table 1:** Mean values and standard deviations of the total time of wound healing for the control and test groups

**Cells**	**Groups**	**Mean (days)**	**SD**
**NOF**	0.1% E-liquid	6.17	0.75
	1% E-liquid	7.33	0.51
	5% E-liquid	9.167	0.75
	10% E-liquid	12.33	1.03
	Control (DMEM)	5.17	0.41
**OKF6/ TERT2**	0.1% E-liquid	6.00	0.63
	1% E-liquid	6.67	0.52
	5% E-liquid	7.67	0.52
	10% E-liquid	10.50	0.84
	Control (DMEM)	5.05	0.63

## Discussion

Three different cell types, including NOFs, OKF6/TERT2 keratinocytes, and cancerous TR146 keratinocytes, were used in this study. Normal oral fibroblasts were selected as they are thought to play a major role in mucosal wound healing; they are also responsible for extracellular matrix synthesis in the connective tissue layer. OKF6/TERT-2 keratinocytes exhibit high reproducibility, avoid batch-to-batch variations; they were selected to represent normal oral epithelial cells. Conversely, squamous cell carcinoma-derived TR146 cells were utilized to assess the effects of E-liquid on oral cancer cells.

E-liquid with no added flavors was used to eliminate the confounding effects of different additives that are observed with various types of flavoring agents used in E-cigarettes. Different studies have shown that certain flavors, such as menthol, cinnamon, caramel, butterscotch, bubble-gum, and coffee, have more cytotoxic effects on cells compared to some other flavors.^39-41^

Four different concentrations (0.1%, 1%, 5%, and 10%) of E-liquid solution were prepared to assess the effects of various concentrations of E-liquid on the cells, covering the range from light vapers to heavy e-cigarette users. The duration of exposure included three days and seven days to simulate short-term and medium-term vaping.

In this study, electronic cigarette liquid exhibited an adverse effect on the viability of normal oral fibroblasts and OKF6/TERT-2 keratinocytes. These results are consistent with a previous study that tested different types of E-cigarette liquids on the human periodontal ligament fibroblasts and showed a reduction in the viability of cells in the samples that were exposed to E-liquids.^39^

Conversely, a dose-dependent stimulatory effect of E-liquid on the growth of cancerous TR146 cells was observed in our study, exhibiting increased viability and proliferation of TR146 cells with increasing concentrations of E-liquid. Similarly, histological evaluation of the 3D oral mucosa models showed an increase in the thickness of the cancerous epithelial layer exposed to high concentrations of E-liquid. This is the first study reporting the use of a full-thickness 3D tissue-engineered oral mucosal model for the biological evaluation of electronic cigarettes on cancerous oral tissues. These findings have not been reported previously and might indicate tumor-promoting effects of the ingredients of the E-liquid tested in this study.

Previous studies have raised some concerns regarding the potential effects of nicotine on promoting tumors in the lungs through various possible mechanisms, such as cell migration, proliferation and angiogenesis.^20-21^

The influence of nicotine on dysplastic oral keratinocyte cell line and precancerous lesions of the mouse tongue has been investigated previously, showing an inhibitory effect on apoptosis and a stimulatory effect on the growth of oral precancerous lesions.^44^ Similarly, Chernyavsky et al^42^ assessed the tumor-promoting effects of nicotine on oral and lung cancer cells. Nicotine exhibited resistance to apoptosis, increasing the counts of both lung and oral cancer cells.

Some other studies have used monolayer cell culture systems to assess the cytotoxicity of electronic cigarettes.^39-43^ Different types of cell lines have been exposed to either electronic cigarette aerosols or E-liquid solutions. These studies have also confirmed the adverse effects of E-cigarettes, with some cell lines being more sensitive than the others.

Recently, studies have been conducted to assess the effects of electronic cigarettes on oral mucosal cells. In a study by Yu et al,^43^ electronic cigarette exposure reduced cell viability along with high levels of apoptosis and necrosis, with alterations in augmented DNA strands, in normal epithelial and squamous cell carcinoma (head and neck) cell lines. However, Guttenplan et al^44^ observed a stimulatory effect of E-cigarette exposure on the proliferation of human oral leukoplakia cells. Conversely, Willershausen et al^37^ reported a reduction in cell proliferation of periodontal ligament fibroblasts after exposure to various E-liquids.

Sundar et al^45^ utilized human periodontal ligament fibroblasts and a 3D gingival epithelium-only tissue model to assess the effects of electronic cigarettes, reporting an increase in pro-inflammatory cytokines and an elevated oxidative/carbonyl stress resulting in the production of high levels of cyclooxygenase-2 and prostaglandin E2. Although the 3D split-thickness tissue model used in this study was more clinically relevant than monolayer cell culture systems, it lacked the connective tissue component.

A study by Sancilio et al^46^ raised concerns regarding E-cigarette’s role in the pathogenesis of oral diseases. Human gingival fibroblasts exposed to E-Liquids showed decreased production of collagen I and increased levels of lactate dehydrogenase.

There is a lack of research on the effects of E-cigarettes on human oral mucosa wound healing. It has been indicated that exposure to E-cigarettes reduces the viability of cells and compromises cell migration.^46^ Therefore, this study aimed to investigate the effects of E-cigarette liquids with different concentrations on oral mucosa wound healing. Two wound healing models were used in this study based on normal oral fibroblasts to represent connective tissue wound healing and OKF6/TERT2 keratinocytes to simulate epithelial wound healing. The results of this in vitro assay were consistent with the results of the cytotoxicity tests and indicated that the E-liquid tested in this study might have potential dose-dependent adverse effects on oral mucosa wound healing, prolonging the healing times both in the epithelial and the connective tissue layers. In a recent study,^47^ human gingival fibroblasts were exposed to three different groups (cigarette smoke condensate, nicotine-free or nicotine-rich electronic cigarette vapor condensates), and the results showed that both cigarette smoke and electronic cigarette vapors affected the proliferation and migration of fibroblasts. Additionally, the cell scratch test revealed delayed wound healing, consistent with the findings of this study.

There were certain limitations in our study; firstly, this was an in-vitro study, and the results may not be extrapolated to an in-vivo situation. Secondly, only E-liquids were tested in this study rather than the E-cigarette aerosols. Ideally, the cytotoxic effects of E-cigarettes should be assessed in both liquid and vapor form, as the E-cigarette vapors come into direct contact with oral mucosa.

Hence, further research is required to overcome these limitations by assessing E-cigarettes of different flavors and various concentrations of nicotine. The liquid, as well as the vapor form of E-cigarettes, can be tested and compared with the conventional cigarette smoke. Furthermore, evaluating the long-term effects of E-cigarettes would further add to our understanding of the biological effects of E-cigarettes on human oral tissues.

## Conclusion

This in vitro study revealed that short-term and medium-term exposure to the electronic cigarette liquid had dose-dependent cytotoxic effects on normal human oral fibroblasts and OKF6/TERT-2 oral keratinocytes. However, E-liquid exposure had a cumulative stimulatory effect on the growth of cancerous TR146 keratinocytes using both monolayer and 3D cell culture systems.

The full-thickness 3D tissue-engineered human oral mucosal model has the potential to be used as a clinically relevant biological test system for the evaluation of electronic cigarettes.

In addition, E-liquid exposure prolonged the wound healing of both normal oral fibroblasts and OKF6/TERT-2 epithelial cells.

## Competing Interests

The authors declare no conflict(s) of interest related to the publication of this work.

## Authors’ Contributions

Conceptualization, Z.S., A.A., T.A., K.F., L.T. and K.M.; Data curation, Z.S. and A.A.; Investigation, Z.S., A.A., T.A., K.F., L.T. and K.M.; Methodology, Z.S., A.A., T.A. and K.F.; Supervision, L.T. and K.M.; Validation, Z.S., and A.A.; Writing—original draft, Z.S. and A.A.; Writing—review and editing, T.A., K.F., L.T. and K.M.

## Ethics Approval

This study has been approved by National Research Ethics Service, NRES Committee London—Hampstead. Research Ethics Committee (REC) Reference number: 15/LO/0116; date of approval: 21/01/2015.
